# Case Report: Two Cases of Pulmonary Artery Dissection in Young Infants

**DOI:** 10.3389/fcvm.2022.872049

**Published:** 2022-03-25

**Authors:** Chunnian Ren, Libing Zhang, Huan Yan, Zhengbing Yang

**Affiliations:** Department of Cardiothoracic Surgery, School of Medicine, Chengdu Women's and Children's Central Hospital, University of Electronic Science and Technology of China, Chengdu, China

**Keywords:** pulmonary artery dissection, young infants, treatment, surgery, case report

## Abstract

Pulmonary artery dissection (PAD) is a rare disease. This article reports the treatment of PAD in young infants for the first time. Both cases of the infants were treated with surgery. Different surgical methods achieve different results, which provide ideas for treating PAD in young infants.

## Introduction

Helmbrecht et al. was the first to report a case of pulmonary artery dissection (PAD) in 1842 (1). A recent literature review reported 150 cases of patients with PAD (1), but all were concentrated on adults. Here, we report two cases of young infants diagnosed with PAD. Surgical treatment was performed concurrently, and good results were achieved.

## Clinical Summary

### Case 1

**Figure 1:**
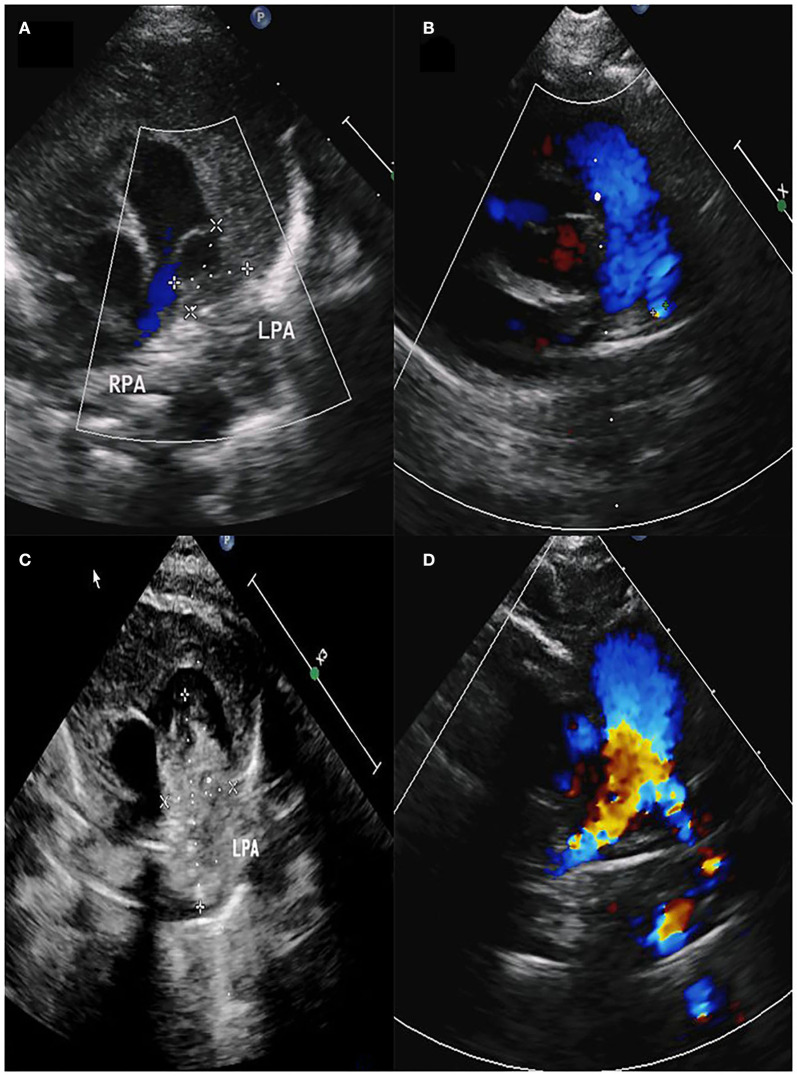
**(A)** Space-occupying lesion with a size of 11.6 × 10.5 mm in the pulmonary artery. **(B)** Diameter of the left pulmonary artery was 2.2 mm. **(C)** Space-occupying lesion with a size of 31.9 × 15.7 mm in the pulmonary artery. **(D)** Diameter of the left pulmonary artery was 3.9 mm.

**Figure 2:**
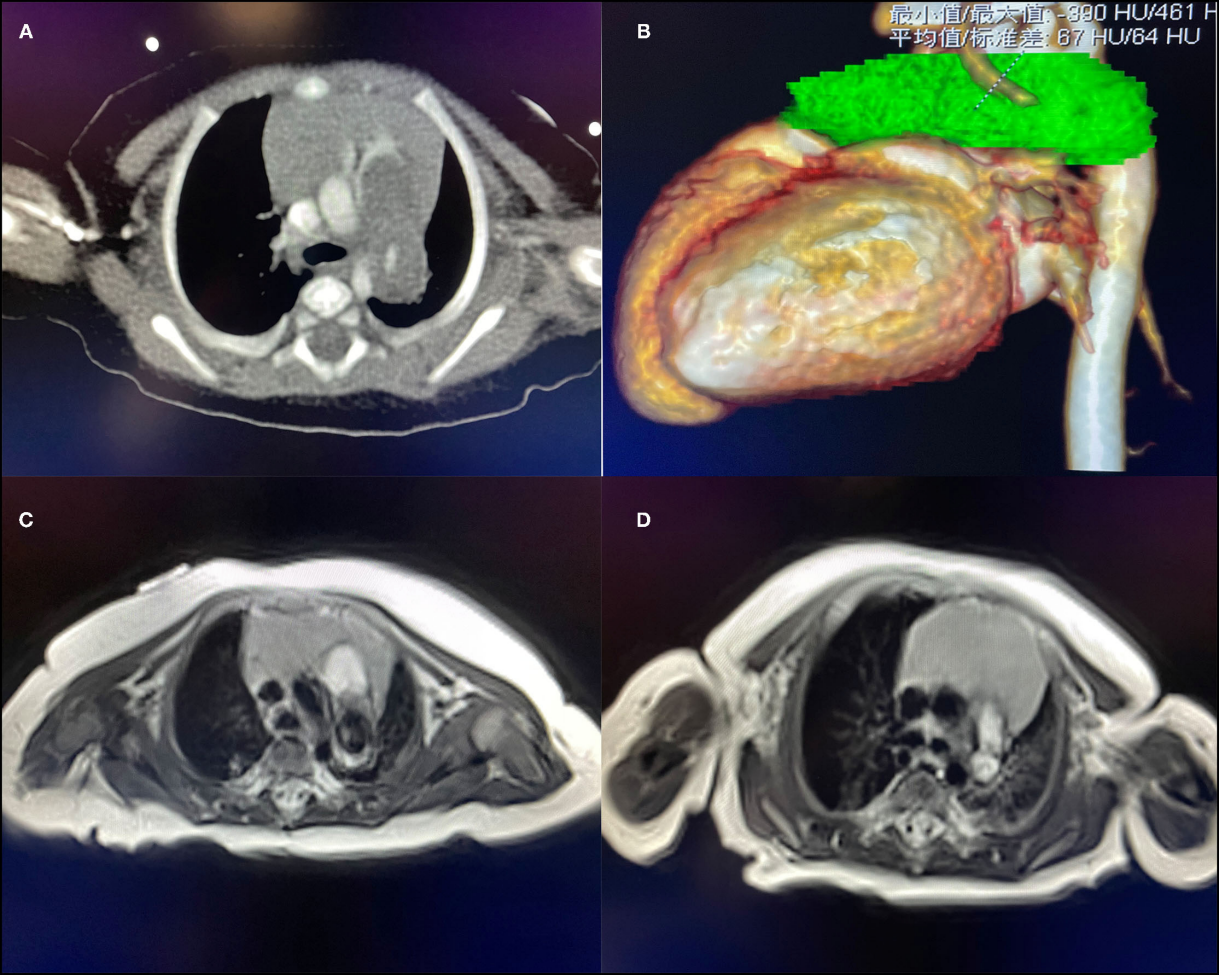
**(A)** CT suggests space-occupying lesions of the pulmonary artery. **(B)** The green part is the three-dimensional imaging of the lesion. **(C)** T1 image of the MRI of case 1 found a pulmonary artery space-occupying lesion. **(D)** T1 image of the MRI of case 2 found a pulmonary artery space-occupying lesion.

Male, 57 days old, admitted to hospital because of pneumonia; there was no abnormality in the pregnancy checkup of the mother. No other deformities were found. On admission, the measured weight was 5.2 kg, length was 57 cm, and a grade 3/6 systolic murmur in the pulmonary valve area was heard, but no thrill was heard. Cardiac color Doppler ultrasound showed a space-occupying lesion in the trunk of the pulmonary artery to the start of the left pulmonary artery. The space-occupying lesion causes obvious stenosis of the left pulmonary artery lumen (Figure 1A), the diameter of the left pulmonary artery is 1.8 mm, and maximum blood flow velocity is 2.3 m/s. Cardiac CT (computed tomography) examination revealed space-occupying lesions in the trunk of the pulmonary artery and the start of the left pulmonary artery area, but the nature of the lesions is unclear (Figures 2A,B). Cardiac MRI (magnetic resonance imaging) showed space-occupying lesions in the trunk of the pulmonary artery and the start of the left pulmonary artery area; therefore, the possibility of hematoma or mural thrombosis was considered (Figure 2C).

**Figure 3:**
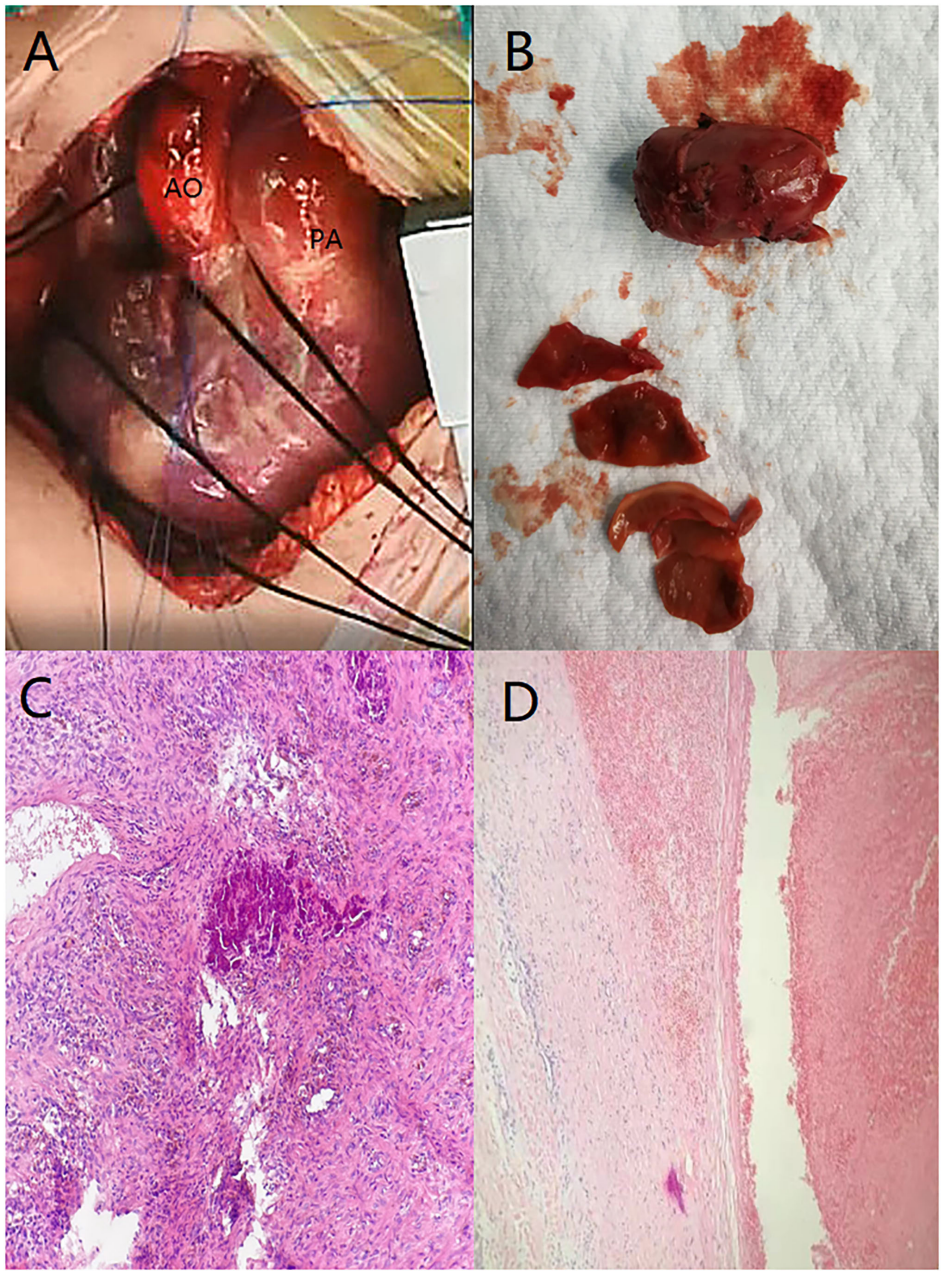
**(A)** Anterior wall of the trunk of the pulmonary artery was enlarged. **(B)** Thrombus-like tissue was removed during the operation, the color is darker, and the blood vessel wall is visible. **(C)** Visible dark calcification and surrounding brown-yellow hemosiderin changes. **(D)** Static image shows that the membrane is separating.

After anesthesia, a median incision was made to open the chest, and cardiopulmonary bypass was established. During the operation, the pulmonary artery was significantly dilated, and local bulging was observed. A thrombotic tissue was seen in the anterior wall of the trunk of the pulmonary artery extending to the start of the left pulmonary artery, and the thrombus-like tissue is about 2 cm long and 1 cm in diameter (Figures 3A,B). The aortic end of the ductus arteriosus was closed and fibrotic. The abnormal anterior wall of the pulmonary artery was removed, the aortic end of the arterial duct was sutured and ligated, and pulmonary angioplasty was performed with an autologous pericardial patch up to the start of the left pulmonary artery. No full-segment left pulmonary artery patch widening was performed.

Postoperative pathological findings: PAD, mural thrombus was seen, and membrane separation was seen (Figure 3D). The pathological section showed presence of hemosiderin and calcification in the diseased tissue (Figure 3C). Cardiac color Doppler ultrasound was reviewed 3 months after the operation. The diameter of the right pulmonary artery was 4 mm, the diameter of the left pulmonary artery was 2.2 mm, and the maximum velocity of blood flow was 1.84 m/s (Figure 1B).

### Case 2

Female, 60 days old, measured weight was 5 kg, and length was 55 cm. She was admitted to hospital because of recurring pneumonia. A grade 3/6 systolic murmur in the pulmonary valve area was heard on admission, but no thrill was heard. No other deformities were found. Cardiac color Doppler ultrasound screening revealed a space-occupying lesion in the trunk of the pulmonary artery to the start of the left pulmonary artery (Figure 1C), filamentous blood flow was seen in the left pulmonary artery, and maximum blood flow velocity was 3.06 m/s. Cardiac MRI showed space-occupying lesions in the trunk of the pulmonary artery and the start of the left pulmonary artery area (Figure 2D). PAD was seen during surgery, and a thrombotic tissue was seen in the anterior wall of the trunk of the pulmonary artery extending to the start of the left pulmonary artery. The thrombus-like tissue is about 2 cm long and 1.2 cm in diameter, the aortic end of the ductus arteriosus was closed and fibrotic. Unlike case 1, the pericardial patch expanded the trunk of the pulmonary artery up to the start of the left pulmonary artery; therefore, an autologous pericardium patch was used to widen the left pulmonary artery to the hilum. The color Doppler ultrasound was rechecked 3 months after the operation. The right pulmonary artery diameter was 4.9 mm, the diameter of the left pulmonary artery was 3.9 mm, and the maximum velocity of blood flow was 2.19 m/s (Figure 1D).

## Discussion

PAD was once considered to be a rare disease with a high fatality rate (2). However, reports were all concentrated among adults r, and there was no specific report on PAD in young infants.

The majority of PADs occurs in the presence of medial degeneration with fragmentation of elastic fibers and generalized dilatation of the pulmonary arterial tree caused by chronic pulmonary hypertension (3). In a small number of cases, PAD may occur in the site of local aneurysm formation. Aneurysms are most commonly associated with congenital heart disease that causes persistently high pulmonary flow velocity and pulmonary hypertension, and, especially, hemodynamic changes caused by persistent arterial ducts, leading to increased pulmonary blood flow, increased intravascular pressure, and increased wall stress (3). We reported two patients who were both infants and had no pulmonary hypertension and other underlying diseases. Closed ductus arteriosus was seen during the operation, considering that PAD may be related to continuous acceleration of blood flow at the ductus arteriosus during the fetal period that led to increased intravascular pressure and tearing of the intima. The postoperative pathology shows that the membrane is separating and further confirms this conjecture. The pathological section showed presence of hemosiderin and calcification in the diseased tissue, indicating that the formation of the diseased tissue took a long time. It is further speculated that the formation of PAD may be related to PDA during the fetal period.

Adults with PAD most often present with chest pain, dyspnea, and hemoptysis (2). Unlike in adults, PAD in young infants usually manifests as recurring pneumonia. The two patients we reported came to the hospital for recurring pneumonia. The first infant we reported underwent CT examination. However, because of rare experience in diagnosing PAD in young infants, radiologists finally considered it as a space-occupying lesion, but the nature of the lesion is unclear. At the same time, an MRI examination was also performed to confirm the diagnosis. The final diagnosis of the MRI was hematoma or mural thrombus. However, in the end, the patient was diagnosed with PAD, which was further confirmed by intraoperative findings and postoperative pathology. Although CT is considered the best method for diagnosing PAD (2), for PAD in young infants, MRI can provide more information through a multi-level comparison. At the same time, compared to CT examinations, MRI examinations can avoid the use of contrast agents on young infants.

In our report, the lesions in the two infants were present in the trunk of the pulmonary artery to the start of the left pulmonary artery. For the first infant, the anterior wall of the diseased pulmonary artery was removed, and an autologous pericardial patch was used for pulmonary angioplasty. Although the postoperative review showed that the operation was good, the left pulmonary artery was still narrower than usual. Considering that infants are in the stage of growth and development, PAD compresses the left pulmonary artery, resulting in long-term obstruction of blood flow to the left pulmonary artery, which further affects the development of pulmonary blood vessels. Eventually, it causes irreversible stenosis of the left pulmonary artery. The same procedure used for the first infant was adopted for the second infant. At the same time, we completely freed the left pulmonary artery and used a pericardial patch to expand the left pulmonary artery to the left hilum. The postoperative review revealed that the second patient had no left pulmonary artery stenosis. Because there is no previous report on surgical methods for infants with PAD, our center has reported on a surgical procedure for infants with PAD. We suggest that using an autologous pericardium patch is feasible to expand the pulmonary artery for PAD in young infants. At the same time, for the left and right pulmonary artery branches with lesions, although lesions only exist at the start of the left or the right pulmonary artery, full-segment expansion of the pulmonary artery branch should be performed during surgery to avoid left or right pulmonary stenosis.

## Data Availability Statement

The original contributions presented in the study are included in the article/Supplementary Material, further inquiries can be directed to the corresponding author.

## Author Contributions

CR wrote the manuscript. ZY, HY, and LZ helped in the clinical case analysis. All the authors contributed in the care, diagnosis, and treatment of the patients.

## Conflict of Interest

The authors declare that the research was conducted in the absence of any commercial or financial relationships that could be construed as a potential conflict of interest.

## Publisher's Note

All claims expressed in this article are solely those of the authors and do not necessarily represent those of their affiliated organizations, or those of the publisher, the editors and the reviewers. Any product that may be evaluated in this article, or claim that may be made by its manufacturer, is not guaranteed or endorsed by the publisher.

## Abbreviations

PAD: pulmonary artery dissection
CT: computed tomography
MRI: magnetic resonance imaging
PDA: patent ductus arteriosus.
